# Expectations of iPad Use in an Internal Medicine Residency Program: Is It Worth the “Hype”?

**DOI:** 10.2196/jmir.2524

**Published:** 2013-05-08

**Authors:** Nancy Luo, Christopher G Chapman, Bhakti K Patel, James N Woodruff, Vineet M Arora

**Affiliations:** ^1^Department of MedicineUniversity of ChicagoChicago, ILUnited States; ^2^Associate Program Director, Internal Medicine ResidencyDepartment of MedicineUniversity of ChicagoChicago, ILUnited States

**Keywords:** iPad, mobile tablet computing, technology, expectation dynamics, hype

## Abstract

**Background:**

While early reports highlight the benefits of tablet computing in hospitals, introducing any new technology can result in inflated expectations.

**Objective:**

The aim of the study is to compare anticipated expectations of Apple iPad use and perceptions after deployment among residents.

**Methods:**

115 internal medicine residents received Apple iPads in October 2010. Residents completed matched surveys on anticipated usage and perceptions after distribution 1 month prior and 4 months after deployment.

**Results:**

In total, 99% (114/115) of residents responded. Prior to deployment, most residents believed that the iPad would improve patient care and efficiency on the wards; however, fewer residents “strongly agreed” after deployment (34% vs 15% for patient care, *P*<.001; 41% vs 24% for efficiency, *P*=.005). Residents with higher expectations were more likely to report using the iPad for placing orders post call and during admission (71% vs 44% post call, *P*=.01, and 16% vs 0% admission, *P*=.04). Previous Apple iOS product owners were also more likely to use the iPad in key areas. Overall, 84% of residents thought the iPad was a good investment for the residency program, and over half of residents (58%) reported that patients commented on the iPad in a positive way.

**Conclusions:**

While the use of tablets such as the iPad by residents is generally well received, high initial expectations highlight the danger of implementing new technologies. Education on the realistic expectations of iPad benefits may be warranted.

## Introduction

The use of mobile technology in hospitals is not a new development. Despite many iterations of PDAs and tablet computers, medical professionals have traditionally been stymied by poor user interfaces, inadequate information density, and insufficient interoperability [1]. The introduction of the Apple iPad sparked tremendous excitement. Reports quickly surfaced about the iPad being used in operating rooms and emergency rooms [2-4]. The Stanford and Yale medical schools adopted iPads for use as an adjunct to their medical curriculum [5,6]. Residency programs, including one at the University of Chicago, adopted the iPad. Systematic analyses of physician order entry from the electronic medical record demonstrated that the iPad affords significant improvements in both perceived and actual efficiency [7]. Other reviews confirm utilization with regard to communication and information management among providers in a health care setting [8].

**Figure 1:**
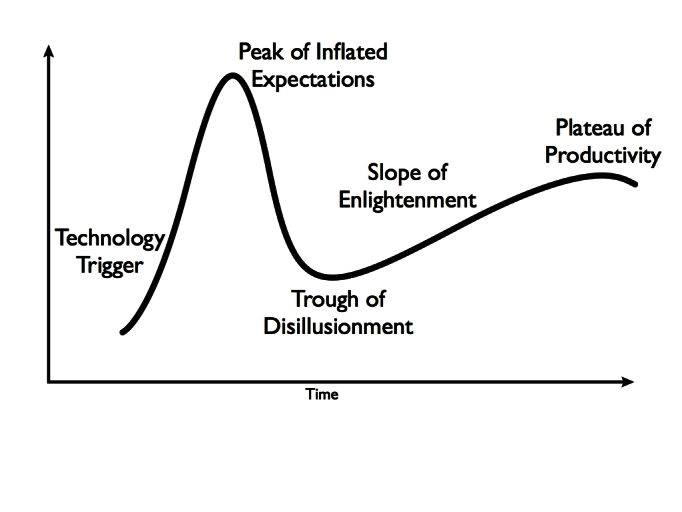
Gartner hype cycle.

While the release of the iPad was associated with tremendous excitement, any new technology can be plagued by inflated expectations, leading inevitably to disappointment. The Gartner Hype Cycle model describes the change in maturity and adoption rate for emerging technology over time and suggests any burgeoning technology is subject to the effects of hype [9]. As buzz grows after the release of a new technology, early adopters surface with anecdotal stories of tremendous success. Soon thereafter, as early expectations are not fully realized, popular sentiment swings the other direction, creating a “trough of disappointment”. Figure 1 shows a graphical representation of the excitement, adoption, and mature application of new technologies plotted over time.

Technology adoption will only become well embedded if new uses are realized, tested, and implemented, thereby permitting mainstream users to adopt the technology and reach the “plateau of productivity”. The Gartner Hype Cycle is facilitated by “early adopters,” or individuals who enter the cycle early, near the technology trigger. Early adopters are often instrumental in spreading the use of the technology to mainstream users. Mainstream users adopt the technology only once it has proven that its benefits outweigh the costs of learning a new interaction [10]. This model highlights the importance of characterizing early adopters who can become champions of new technology to help realize its full adoption.

In the hospital setting, it is critical to accurately assess whether the introduction of the iPad is associated with hype or inflated expectations, and how this hype affects adoption of the device. Furthermore, it is also important to understand characteristics of early adopters who can spread the innovation to facilitate large-scale adoption. Therefore, the objective of our study is to (1) characterize the hype associated with iPads by comparing anticipated use of an iPad with actual use among residents, and (2) characterize whether personal adoption of iPads is associated with various factors including prior Apple usage, year in training, and gender.

## Methods

### iPad Deployment

In October 2010, all 115 internal medicine residents at the University of Chicago Medical Center were given Apple iPads. An initial task force of Internal Medicine faculty, residents, and department administrative staff, which had convened to assess and manage iPad distribution, had already determined through a small 2-month pilot study of 5 residents that iPads had the potential to improve house staff workflow on the wards. The pilot established the benefits of a carrying strap while using the iPad during daily rounds as well as the need to improve wireless network reception and coverage. Before being issued to a resident, each iPad was set up with access to the following software: (1) MobileIron, an institutionally controlled device management application that personalized and managed the permissions and user restrictions on the device, (2) Epic electronic health record and computer provider order entry systems via Citrix client, (3) short links to both the electronic paging directory and the institutional subscription to UpToDate, and (4) Epocrates, a mobile application drug database. Upon receiving the device, all residents attended a 1-hour orientation session covering its appropriate clinical use.

### Study Design

This was a matched pre/post cohort survey study, using combined paper and online surveys (Perseus) to ensure a high response rate. To match responses, all respondents created their own unique identifier that enabled pairing of pre- and post-use responses but rendered the data anonymous. Residents were surveyed in the month prior to receiving their iPads with completion of the survey required prior to receiving an iPad. After 4 months of use in the hospital, residents were given the post-use survey. We felt a 3-month learning curve would sufficiently encompass most people’s ability to learn and incorporate the iPad into their workflow. This study was deemed exempt from review by University of Chicago Medicine Institutional Review Board.

### Data Collection

The authors developed the survey items to assess demographics, hype, and usage of the iPad. In addition to the standard demographic questions, such as age, gender, and training year, the pre-use survey included questions about current barriers to efficiency, attitudes toward the iPad, and expectations on its utility in the workplace. The post-use survey measured iPad effects on workflow and efficiency as well as self-reported estimates of device usage in various clinical scenarios such as rounding, inpatient and outpatient use, teaching, and use in front of patients through a 5-point Likert scale (with 5 being “strongly agree” and 1 being “strongly disagree”).

Hype or anticipated excitement regarding the iPad was assessed on the pre-survey through the resident’s selection of one of four statements with which they most agreed: (1) “I perceive little or no value in adding the iPad to our workflow”, (2) “I would be interested in seeing how the iPad works, but I don’t have a lot of extra time to learn a new system”, (3) “I think the iPad will be great and am excited to use it on the wards”, or (4) “It’s all I need in my white coat! I already have one or... [am] seriously considering buying my own.” The language used was developed by 2 resident champions (NL, CC) to reflect the feeling of the anecdotal emotions that other residents had expressed to them and was reviewed by the rest of the investigator team. A resident who exhibited hype was defined as whether he or she agreed with the latter two statements expressing positive expectation or excitement. Residents also identified various work-related tasks they performed with the iPad such as entering orders, typing notes, or reviewing labs and imaging.

### Data Analysis

Descriptive statistics were performed to summarize the percentage of residents that reported demographic characteristics (gender, postgraduate year [PGY]), prior Apple iOS operating system use, perception of hype, and perceived and actual usage of the iPad for various tasks. Usage items were dichotomized to reflect those residents that reported frequent use of the iPad to perform a task. Two-sample tests of proportion were used to test for differences before and after iPad deployment between perceived and actual usage. To test the association between reporting hype (pre-use survey) and actual usage of the iPad (post-use survey), pre- and post-data were merged using the participant’s unique identifier, which enabled a chi-square test. This merged dataset also enabled analysis of the association between demographic characteristics (pre-use survey) and adoption of the iPad (post-use survey) using chi-square tests. All statistical tests were performed in Stata 11.0, with statistical significance defined as *P*<.05.

## Results

All of the residents except one completed both surveys (115/115 responses in the pre-use survey and 114/115 responses in the post-use survey). One resident returned his iPad to the program after 1 month of use. Of the 114 respondents in the post-use survey, 45 (39.5%) were PGY-1, 32 (28%) were PGY-2, and 37 (32%) were PGY 3-4. Of the residents, 55 (48%) were female, and 45 (39.5%) already owned a personal Apple product with a similar operating system to the iPad, like iPhone, iPod Touch, or a personal iPad. Hype was generally high, with 79% (90/114) of residents reporting excitement for the iPad. Interns had statistically higher excitement about the iPads than their peers: 91% (41/45) PGY-1, 78% (25/32) PGY-2, 65% (24/37) PGY-3, *P*=.02 (Table 1).

**Table 1:** Percentage of residents displaying hype.

Characteristics		Percentage
**Apple user**		
	Yes	83% (39/47)
	No	76% (51/67)
**PGY**		
	PGY-1	91% (41/45)^a^
	PGY-2	78% (25/32)^a^
	PGY-3	65% (24/37)^a^
**Gender**		
	Male	78% (46/59)
	Female	80% (44/55)
**All residents**		
		79% (90/114)

^a^ Difference between PGY is statistically significant using chi-square test (*P*=.02).

**Figure 2:**
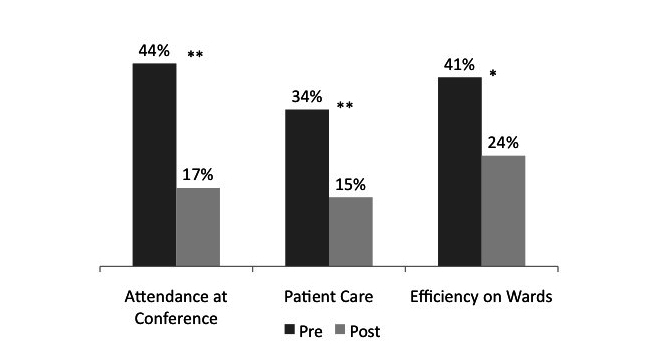
Expectations of iPad use. *P=.005, 2-sample tests of proportion. **P<.001, 2-sample tests of proportion.

Prior to using the iPad, residents expected that the iPad would have positive benefits on their daily practice, with several at the extremes. For example, several residents reported “strong” agreement with the potential impact of the iPad on future attendance at conference (44% or 50/114), benefits to patient care (34% or 39/114), and increased efficiency on wards (41% or 47/114). However, 4 months after deployment, significantly fewer residents felt that the iPad benefited attendance at conference, patient care, and efficiency on the wards with strong agreement: 17% or 19/114, *P<*.001; 15% or 17/114, *P*<.001; 24% or 27/114, *P*=.005, respectively using 2-sample tests of proportion. Figure 2 shows a decrease in percentage of respondents choosing “I strongly agree that the iPad will improve…” in 3 areas before and after using the iPad (n=114).

**Figure 3:**
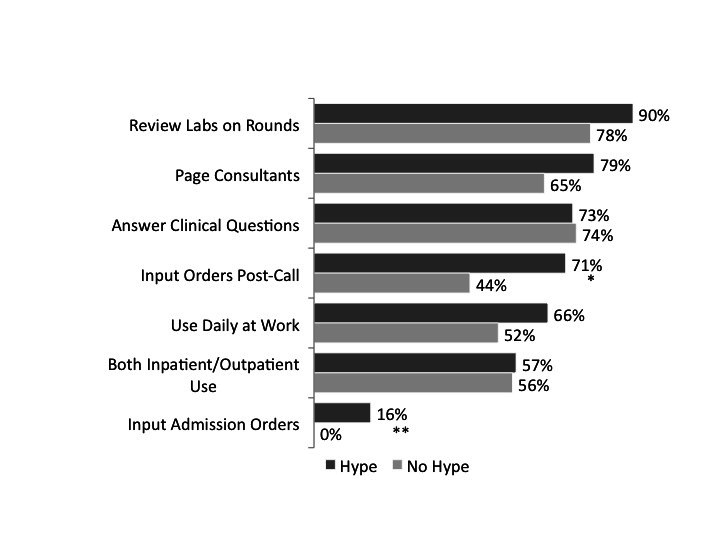
iPad usage by task: Hype versus non-hyped residents. *P=.01, chi-square tests. **P=.04, chi-square tests.

After merging the pre- and post-use data, we excluded 5 individuals whose pre- and post-identifiers did not match, leaving 109 for this analysis. Usage of the iPad was high for many tasks, such as reviewing labs (87% or 95/109), paging (76% or 83/109), and answering clinical questions (73% or 80/109). For these tasks, there was no association between reported hype prior to receipt of the iPad and usage of the iPad after distribution (see Figure 3 for a comparison of use between residents exhibiting hype vs those without hype, n=109). In contrast, residents who reported higher hype before iPad distribution were significantly more likely to report using the iPad for tasks related to placing orders, particularly entering orders post call and during admission: 71% or 61 of 86 hype-residents vs 44% or 10 of 23 nonhype-residents, post call, *P*=.01; and 16% or 14/86 vs 0% or 0/23, admission, *P*=.04, using chi-square tests (Figure 2). Interestingly, after iPad deployment, more residents reported they preferred pen and paper to organize their thoughts than before: 67% (76/114) post vs 39% (44/114) pre, *P*<.001 using chi-square test.

One of the strongest predictors of long-term iPad adoption was whether the resident was a private owner of an Apple iOS product such as iPhone, iPad, or iPod touch. Apple users were significantly more likely than non–Apple users to use the iPad to answer clinical questions (84% or 38 of 45 Apple users vs 65% or 45 of 69 non–Apple users, *P*=.02 using chi-square test) and enter orders post call (78% or 35/45 vs 57% or 39/69, *P*=.02 using chi-square test). Despite interns’ higher initial expectations, they were not more likely to use the iPad than senior residents, with the exception of using the iPad to input orders during rounds (86% or 38/44 vs 64% or 45/70, *P*=.01 using chi-square test). No significant difference was seen in iPad usage by gender.

After the deployment of the iPad, residents did report positive benefits of the iPad, such as less time searching for an available desktop computer and efficiency gains. Most (84% or 96/114) residents thought the iPad was a good investment for the residency program. Moreover, 58% (66/114) reported that a patient had commented on the iPad in a positive way.

## Discussion

### Principle Results

Our study confirms that distribution of the iPad in an internal medicine residency program is associated with high perceived expectations or hype. In fact, 4 months after iPad deployment, actual use of the device for certain tasks fell short of these initial high expectations. Residents who reported more hype prior to iPad deployment were more likely to use the iPad to enter orders. Lastly, those residents who used Apple products prior to iPad deployment were more likely to report higher usage of the iPad.

It is worth exploring why only certain tasks were associated with initial hype. One potential explanation is that many simple tasks (ie, reviewing labs, paging, and answering clinical questions) are inherently easier to learn. In contrast, entering orders post call and placing admission orders through the iPad are difficult, time-consuming tasks. Because these tasks are inherently more complex, it may be that residents who showed more excitement were more willing to expend the effort necessary to use their iPads in situations that required more time investment and effort. On the other hand, residents who did not report hype may have been more likely to revert to traditional methods when faced with technically complex tasks. In developing and encouraging continued use of the iPad and other tablets in the health care environment, it will become important to recognize which tasks are inherently easier to complete on a tablet compared to a desktop computer and which are not. Highlighting the simpler tasks will increase overall use, while efforts should be undertaken with developers to simplify the more difficult tasks.

It is not surprising that interns and Apple users were more likely to demonstrate hype. Interns, because of their age and comfort with technology, and Apple users, because of their existing familiarity with the interface, are more likely to be classified as “early adopters” in technology use [10]. Further characterizing what differentiates interns and Apple users from other groups may lead to methods of encouraging other residents to increase their hype, and accordingly, increase efficiency. Cultivating champions among these groups with high hype may be a helpful strategy to increase use and provide assistance to those residents who are more skeptical or are struggling with their device.

### Comparison With Prior Work

These findings suggest several intriguing implications for other hospital-based physicians. Previously, we demonstrated that iPad deployment led to a higher proportion of orders being placed both earlier in the hospitalization and before the primary team left the hospital, leading to increased efficiency [7]. This effect was demonstrated through EMR order entry data. Here, we observed that when residents experienced greater hype for the iPad, they also placed significantly more orders post call, an area where previous efficiency gains were achieved. It is conceivable that continuing to encourage excitement and set high expectations will create greater gains in efficiency. Indeed, previous research in education has suggested that creating an engaged and excited student population leads to better performance [11]. Conversely, it is also important to understand how and why the iPad may fall short of expectations. Residents’ expressing a stronger preference for pen and paper even after using the tablet for 4 months highlights that mobile computing still lacks certain critical characteristics unique to the analog domain.

### Limitations

There are several limitations to this study. First, this study was conducted in a single institution, with only Apple iPad and no other tablet devices, so results may not be transferable. Results may be skewed towards more inflated expectations because baseline surveys were completed as part of an initiative to receive an iPad. In addition, to measure hype, we created our survey tool since we lacked a validated one. Furthermore, data on how iPads were used were collected via self-report, which could be affected by recall bias.

### Conclusions

In conclusion, this study shows that the deployment of Apple iPads in a residency program was associated with high expectations, consistent with prior models of technology innovation. Careful management of these expectations while promoting champions of use will be necessary to create an environment of successful adoption for mobile technology in the hospital setting.

## Abbreviations

EMR: electronic medical record
iOS: iPhone operating system
PGY: postgraduate year
